# Caffeine and EGCG Alleviate High-Trans Fatty Acid and High-Carbohydrate Diet-Induced NASH in Mice: Commonality and Specificity

**DOI:** 10.3389/fnut.2021.784354

**Published:** 2021-11-22

**Authors:** Xin Xin, Chen Cheng, Cai Bei-yu, Li Hong-shan, Tian Hua-jie, Wang Xin, An Zi-ming, Sun Qin-mei, Hu Yi-yang, Feng Qin

**Affiliations:** ^1^Institute of Liver Diseases, Shuguang Hospital Affiliated to Shanghai University of Traditional Chinese Medicine, Shanghai, China; ^2^Shanghai Key Laboratory of Traditional Chinese Clinical Medicine, Shanghai, China; ^3^Key Laboratory of Liver and Kidney Diseases, Shanghai University of Traditional Chinese Medicine, Ministry of Education, Shanghai, China

**Keywords:** caffeine, EGCG, non-alcoholic steatohepatitis, inflammation, glucose metabolism, lipid metabolism

## Abstract

Caffeine and epigallocatechin-3-gallate (EGCG), which respectively, are the main functional extracts from coffee and green tea, and present protective effects against non-alcoholic fatty liver diseases (NAFLD). These two beverages and their functional extracts are highly recommended as potential treatments for obesity and NAFLD in clinics; however, their pharmacodynamic effects and pharmacological mechanisms in non-alcoholic steatohepatitis (NASH) remain unclear. Therefore, the aim of this study was to explore the commonality and specificity of the pharmacodynamic effects and pharmacological mechanisms of caffeine and EGCG on NASH mice, which were fed with a high-trans fatty acid/high-carbohydrate (HFHC) diet. C57BL/6J mice were fed a normal diet (control group) or an HFHC diet (HFHC group) for 24 weeks. HFHC group mice were additionally treated with caffeine (75 mg/kg) or EGCG (100 mg/kg) for 6 weeks, using obeticholic acid (OCA,10 mg/kg) as a positive control group. The pharmacological effects of the drugs, including effects on glucose and lipid metabolism and liver inflammation and fibrosis, were evaluated. Gene expression in liver tissue samples from the different groups were assessed. Both caffeine and EGCG significantly reduced the liver manifestations of NASH induced by HFHC. The pathological aspects of liver lipid deposition, inflammation, and liver fibrosis in both groups were strongly ameliorated. Of note, most indexes were strongly reversed in the caffeine group, although AST activity, fasting blood glucose, and the HOMA-IR index were improved in the ECGC group. There were 714 differentially expressed genes between the caffeine and HFHC groups and 268 differentially expressed genes between the EGCG and HFHC groups. Twenty and 17 NASH-related KEGG signaling pathways were enriched by caffeine and EGCG. This study confirmed that 75 mg/kg caffeine and 100 mg/kg EGCG could significantly improve liver lipid deposition, glucose metabolism, inflammation, and fibrosis in a mouse model of NASH induced by HFHC. The bioinformatics platform we built for caffeine and EGCG in NASH disease found that the two drugs may greatly overlap in improving the mechanism related to NASH inflammation. However, caffeine may have better potential in regulating glucose metabolism and EGCG may have better potential in regulating lipid metabolism.

## Introduction

Caloric excess and a sedentary lifestyle have led to a global epidemic of obesity and metabolic syndromes (1). Non-alcoholic fatty liver disease (NAFLD), also called metabolic dysfunction-associated fatty liver disease, is a consequence of this epidemic and is estimated to affect up to one-third of the adult population worldwide (2). NAFLD is characterized by excessive cytoplasmic retention of triglycerides (TGs) (3) and ranges from simple steatosis to non-alcoholic steatohepatitis (NASH), cirrhosis, and hepatocellular carcinoma (HCC) (4). The occurrence and development of NAFLD are associated with a variety of factors, including lipid accumulation, increased inflammatory factors, abnormal expression of adipokines, abnormal gut microbiota, genetic predisposition, and oxidative stress (5). Despite ongoing research on NASH/NAFLD, the detailed mechanisms of onset and treatment strategies remain unclear (6). Because no treatment has been approved for NASH, non-pharmacological treatment of NAFLD/NASH is recommended aiming to reduce fatty liver by body weight loss and exercise. Although some drugs and drug combinations, including obeticholic acid, dulaglutide, alogliptin with pioglitazone, and empagliflozin with pioglitazone, are under clinical study (7), these drugs often have insufficient efficacy and are associated with numerous side effects (8). Together with drug therapy for NAFLD, lifestyle factors, especially dietary habits, have been shown to play a central role in the pathogenesis of NAFLD and MetS (9). Recently, there has been great interest in identifying effective compounds from natural sources to treat NAFLD, because these compounds have low toxicity and potentially fewer side effects (10–12).

After water, coffee and tea are the most consumed beverages worldwide (13). Coffee is made from the seeds of the coffee plant, *Coffea*. It is a member of the *Rubiaceae* family and includes hundreds of different species (including the main *arabica* and *robusta*). Caffeine is the main component of coffee. Although coffee and caffeine can increase the risk of cardiovascular disease, numerous studies have demonstrated that coffee and caffeine have hepatoprotective effects against chronic liver diseases (14). Epidemiological and clinical investigations have suggested that the consumption of coffee could reduce the risk of alcoholic liver cirrhosis (15), type-2 diabetes (16), NAFLD (17), and HCC (18, 19), and reduce the progression of NASH, the severity of fibrosis (20), and alanine aminotransferase (ALT) activity in patients with liver injury (21). The recent meta-analysis results of Stefano et al. show that coffee consumption may play a protective role in fibrosis. Both coffee and tea consumption are associated with less likelihood of having metabolicsyndrome (13). A systematic review also showed the protective effect of coffee drinking on severe liver fibrosis in patients with NAFLD (22). A longitudinal study by Chung et al. showed that the increase in coffee consumption was associated with a decrease in the incidence rate of fatty liver in male Korean, suggesting that increased coffee consumption may have protective effects on fatty liver (23). Tea is historically recognized as a typical beverage consumed in Asian countries and has been used for more than 5,000 years in diet and folk medicine (24). Tea is made from the leaves of the *Camellia sinensis* (in the *Theaceae* family) and includes several varieties, such as green and black tea (25). Although teas are consumed for their taste and flavor, several observational studies have highlighted the possible beneficial effects on liver and metabolic health (26). Epigallocatechin-3-gallate (EGCG) is the most abundant polyphenolic catechin in green tea. It has been extensively studied for its anti-inflammatory, anti-cancer, and anti-steatosis health benefits in the liver (27, 28). Recent epidemiological findings have led to *in vitro* and *in vivo* studies on caffeine and EGCG, which provide biological plausibility for their effects (26, 29). Given that coffee and tea are representative beverages in Eastern and Western countries, respectively, there are many studies targeting their main components, caffeine and EGCG, in the treatment of NAFLD. The rat experiment results of Velazquez et al. showed that although low-dose CAF could not reduce hepatic steatosis in lean female rats, CAF at the same dose as green coffee extract could reduce hepatic triglyceride level (30). Wu et al. suggested that EGCG can alleviate HFD-induced NAFLD through inhibition of apoptosis and promotion of autophagy possibly via the ROS/MAPK pathway (31). Du et al. validated the salubrious effects of EGCG on NAFLD and sheds light on a novel mechanistic contribution of EGCG, namely hepatic M1-to-M2 macrophage polarization (32). Tang et al. showed that EGCG could not only reduce the oxidative stress caused by NAFLD, but also improve lipid metabolism, in inflammation cascades, fibrotic response, and HCC tumorigenesis (33). However, there are few studies comparing caffeine and EGCG, and the pharmacological mechanisms by which they exert their effects in the treatment of NAFLD/NASH.

In the present study, we used a high trans fatty acid/high-carbohydrate (HFHC) diet-induced NASH mouse model to compare the effects of caffeine and EGCG on liver fat metabolism, glucose metabolism, inflammation, and liver fibrosis. In addition, we used transcriptomic gene microarray technology to perform a comparative analysis of the pharmacological mechanisms of caffeine and EGCG. Our results may provide a strong basis for the clinical application of caffeine and EGCG-related beverages and biopharmaceuticals in NAFLD/NASH treatment.

## Materials and Methods

### Drug Preparation and Identification

**Figure 1:**
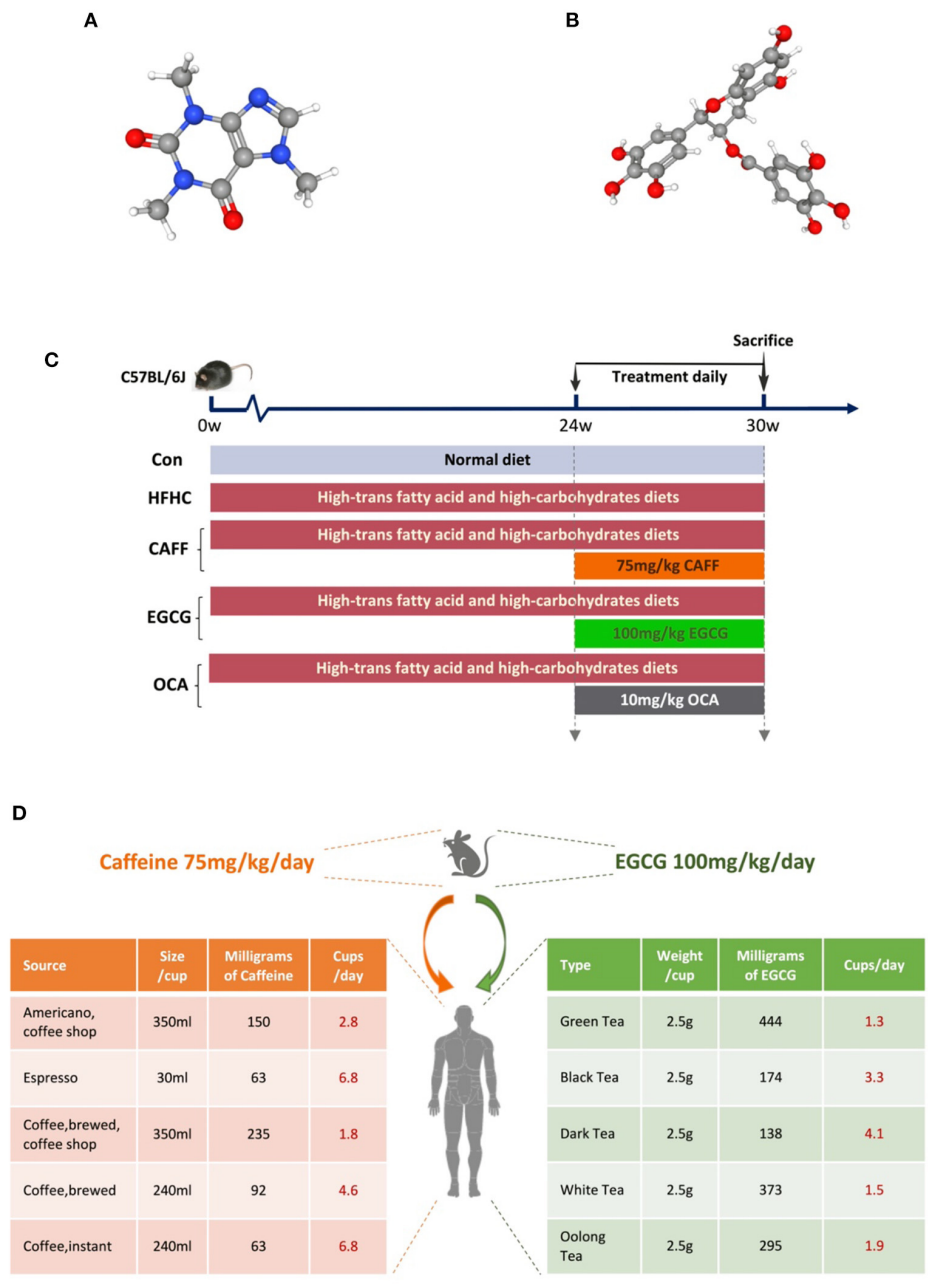
Experimental drugs and flow chart of animal experiment. **(A)** The chemical structure of caffeine. **(B)** The chemical structure of (-) Epigallocatechin gallate. **(C)** The schedule of NASH model modeling and treatment, including the type of diet and the time and dose of treatment intervention. **(D)** The doses of caffeine and EGCG mice were equivalent to the number of coffee or tea cups consumed by humans according to the body surface.

Caffeine (C_8_H_10_N_4_O_2_, molecular weight: 194.19, drug purity 99.75%, CAS 58-08-2, lot number 180526) and EGCG (C_22_H_18_O_11_, molecular weight: 458.38, drug purity 99.03%, CAS: 989-51-5, lot number 180120) were purchased from Shanghai Winherb Medical Technology Co., Ltd. (Shanghai, China). The chemical structures of caffeine and EGCG are shown in Figures 1A,B. OCA was purchased from Biovison (USA, lot number 3J28B18980). The quality control information for all drugs is provided in Supplementary Figure 1.

### Animals and Treatment

Six-week-old male C57BL/6J mice were obtained from Shanghai Slake Experimental Animal Co., Ltd. (Shanghai, China). Mice were housed at 18–24°C at 40–70% relative humidity, with a 12-h light/dark cycle. They were allowed free access to water and food during a 1-week acclimation period. The experimental procedures were conducted in accordance with the National Institutes of Health guidelines for the use of experimental animals. All experimental protocols were approved by the Animal Studies Ethics Committee of Shanghai University of Traditional Chinese Medicine (Permission SZY201710017).

After acclimation for 1 week, mice were randomly divided into a control group (Con, *n* = 8) and an HFHC diet group (HFHC, *n* = 32). The mice in the HFHC group were fed a high-trans-fat diet [58 kcal% fat w/sucrose Surwit Diet (D12331, lot number: 17082101A10, Research Diets, USA)] combined with water containing high carbohydrates (55% fructose + 45% sucrose, concentration 42 g/L) to establish an animal model of NASH. Mice in the Con group were fed a control diet [11 kcal% fat w/cornstarch Surwit Diet (D12328, lot number: 17100212A4, Research Diets)] and normal water. After 24 weeks, mice in the HFHC group were randomly but equally divided into the HFHC group, the caffeine group, the EGCG group, and the OCA group. OCA group was used as the positive control group. Caffeine [75 mg/kg per day (34)], EGCG [100 mg/kg per day (35)], and OCA [10 mg/kg per day (36)] were dissolved in drinking water and administered by gavage once daily for the following 6 weeks; mice in the HFHC group received equal volumes of drinking water. All animals were sacrificed for tissue collection at the end of the 30th week. The technology roadmap used in this study is shown in Figure 1C.

### Serum Biochemical Assays

Serum ALT and aspartate aminotransferase (AST) levels were measured using ALT and AST assay kits (Lot number 20180628, Nanjing Jiancheng Bioengineering Institute, Nanjing, China). To assess glucose metabolism, mice were fasted for 12 h, after which ~3 μL of blood was collected from the tail vein, and fasting blood glucose (FBG) was measured using a Roche blood glucose meter (Roche Diagnostic GmbH, Germany). Fasting insulin (FINS) levels in the serum of mice from each group were measured using enzyme-linked immunosorbent assay (Ultra-Sensitive Mouse Insulin ELISA Kit, lot number: 90080, Crystal Chem, USA). The homeostatic model assessment-insulin resistance (HOMA-IR) index was calculated using the following formula: FBG (mM) × FINS (IU/L)/22.5.

### Hepatic Biochemical Assays

Hepatic TG content was measured using a kit (Lot number: 2018080029, Dong'ou Diagnostic Products Co. Ltd., Zhejiang, China), and hepatic hydroxyproline (HYP) content was measured using an HYP assay kit (lot number: 20180630, Nanjing Jiancheng Bioengineering Institute, Nanjing, China), as previously described (37).

### Histological Examination and Assessment

Fixed liver tissue was dehydrated and embedded using a tissue processor (Leica ASP300) and a paraffin embedding station (Leica EG1160). Then, the sections were stained using a hematoxylin and eosin (H&E) staining kit (lot number 20180530, Nanjing Jiancheng Bioengineering) and a Sirius Red Staining Kit (lot number 20180528, Nanjing Jiancheng Bioengineering). Liver tissue was fixed in liquid nitrogen, embedded in ornithine carbamoyl transferase medium, and sectioned at −20 °C at a thickness of 10 μm. The sections were stained using an Oil Red O staining kit (lot number 20180528, Nanjing Jiancheng Bioengineering), as described previously (37). The NASH Activity Score (NAS) is a semi-quantitative scale to predict NASH. A NAS score of <3 can be used to exclude NASH, a score >4 can be used to diagnose NASH, and a score of 3–4 indicates possible NASH. The higher the score, the more active the lesion, and the higher the degree of hepatic steatosis. Fibrosis staging was used to determine the degree of fibrosis in the liver tissue. The fibrosis stages were scored from F0 to F4, as follows: F0, no fibrosis; F1, perisinusoidal or periportal fibrosis; F2, perisinusoidal and portal fibrosis without bridging formation; F3, bridging fibrosis and nodules; and F4, cirrhosis. Image Pro Plus was used to calculate the positive area of the Sirius Red-stained and Masson-stained images. Eight visual fields were taken from each image to calculate the area of liver fibrosis.

### Western Blot Analysis

Liver tissue was homogenized in lysis buffer (150 mM NaCl, 1% Nonidet P-40, 0.1% SDS, 50 mM Tris-HCl pH 7.4, 1 mM EDTA, 1 mM PMSF, and 1× Roche complete mini protease inhibitor cocktail). The supernatants were collected after centrifugation at 10,000 × g at 4°C for 15 min. Protein concentration was determined using a BCA protein assay kit (Beyotime Institute of Biotechnology, Jiangsu, China). Equal amounts of protein were separated by 10% SDS gel electrophoresis under denaturing and non-reducing conditions and transferred to a polyvinylidene difluoride membrane. The membrane was blocked with 5% non-fat milk in TBST at room temperature for 1 h and then incubated with the appropriate primary antibody at 4°C overnight (antibody information is presented in Supplementary Table 1). After three washes in TBST, the blots were incubated with horseradish peroxide-coupled secondary antibody. The signals were visualized using an enhanced chemiluminescence system (Pierce Biotechnology, Inc., Rockford, IL, USA) and recorded in a chemiluminescence imaging system (ChemiScope 3500 mini, Qin Xiang, China).

### Library Preparation and Illumina Hiseq X Ten Sequencing

Libraries were selected for cDNA target fragments of 200–300 bp on 2% low range ultra-agarose, followed by PCR amplification using Phusion DNA polymerase (NEB) for 15 PCR cycles. After quantification with a TBS380 fluorometer, the paired-end RNA-seq sequencing library was sequenced using an Illumina HiSeq X Ten sequencing system (2 × 150 bp read length). The data were analyzed using the free online Majorbio I-Sanger Cloud Platform (www.i-sanger.com).

### Statistical Analysis

Statistical analysis of the data was performed using IBM SPSS Statistics for Windows, version 26.0. The data were expressed as the means ± standard deviation. After analysis of variance, Student's *t*-test was employed to calculate the statistical significance between two groups. Univariate analysis of variance (ANOVA) was used for multi group comparison. For the nonparametric data, the Kruskal-Wallis H-test was used to analyse the significant difference among more than two groups. The hierarchical grouping data were compared using the Radit analysis. A value of *P* < 0.05 was considered to be statistically significant.

## Results

### Caffeine and EGCG Ameliorate the Abnormally Elevated Body Weight, Liver Weight, and Other Basic Indexes in a Mouse Model of NASH

**Figure 2:**
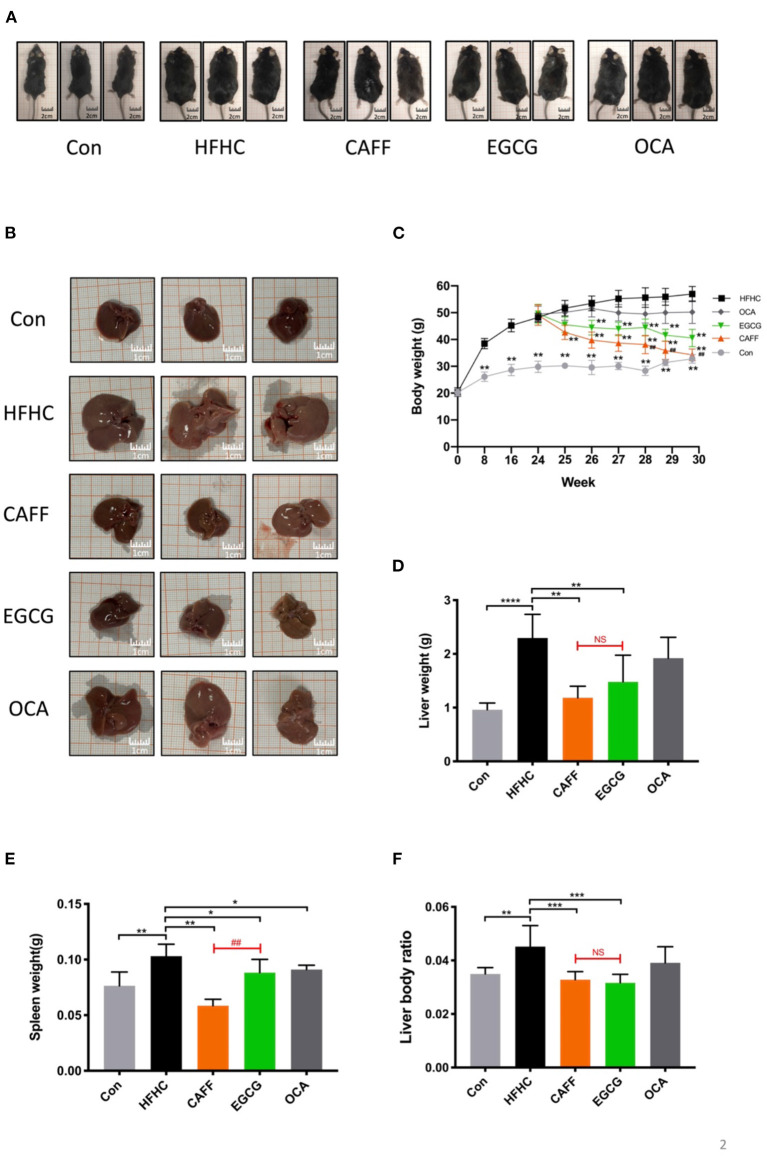
The general condition of mice in each group. **(A)** Representative pictures of mice morphology in each group at sacrifice. **(B)** Representative pictures of liver morphology in each group at sacrifice. Changes in body weight **(C)**, liver weight **(D)**, spleen weight **(E)** and liver body ratio **(F)** of the mice in each group (*n* = 8) vs. HFHC, *P < 0.05, **P < 0.01, ***P < 0.001, ****P < 0.0001; vs. EGCG, ^##^*P* < 0.01, NS, not significant.

We wanted to examine the effects of caffeine and EGCG on the general conditions of mice with NASH. First, we took photos of the general appearance and liver appearance of the mice in each group and observed them with the naked eye (photo background: 1 × 1 cm square paper). The mice in the Con group (*n* = 8) were symmetrical and thin, with a body width of ~3 cm. The mice in the HFHC group (*n* = 8) were obese and round, with a body width of more than 5 cm. Treatment with caffeine or EGCG reduced the obese body shape, and at the end of 30 weeks, the body width of the mice in both the caffeine and EGCG (*n* = 8) treated groups had decreased to ~4 cm. There was no difference between mice in the caffeine and EGCG treated groups to the naked eye. Additionally, there was little difference in body shape of the mice in the OCA (*n* = 8) and HFHC groups (Figure 2A). Similar to the weight trend, at the end of 30 weeks, the livers of the control group mice were dark red and soft. Conversely, the liver from mice in the HFHC group were plump, greasy, hard, and granular on the surface. Yellow and white focal fat deposition could be seen with the naked eye. However, after treatment with caffeine or EGCG, the livers were dark red, significantly reduced in volume, and soft in texture (Figure 2B). In addition, we dynamically examined the body weights of the mice in each group. After 30 weeks, the body weight of the HFHC group increased sharply. Further, there were significant differences in weight between the caffeine and EGCG groups and the HFHC group after 25 weeks. Although the weight of mice in the caffeine and EGCG groups decreased significantly compared with the weight of mice in the HFHC group after intervention, the weight of mice in the caffeine group decreased significantly more than the weight of mice in the EGCG group at the end of 28 weeks (Figure 2C). Similarly, the liver wet weight, spleen weight, and liver/body ratio of mice were significantly lower in the caffeine and EGCG groups than in the HFHC group. Interestingly, the spleen weight of the caffeine group was significantly lower than that of the EGCG group. There were, however, no significant differences in the liver wet weight and liver/body ratio between mice treated with the two treatments (Figures 2D–F). Compared with HFHC, there was only difference in reducing spleen weight in OCA group.

### Caffeine and EGCG Normalize Liver Functional Enzymes and Ameliorate Insulin Resistance in a Mouse Model of NASH

**Figure 3:**
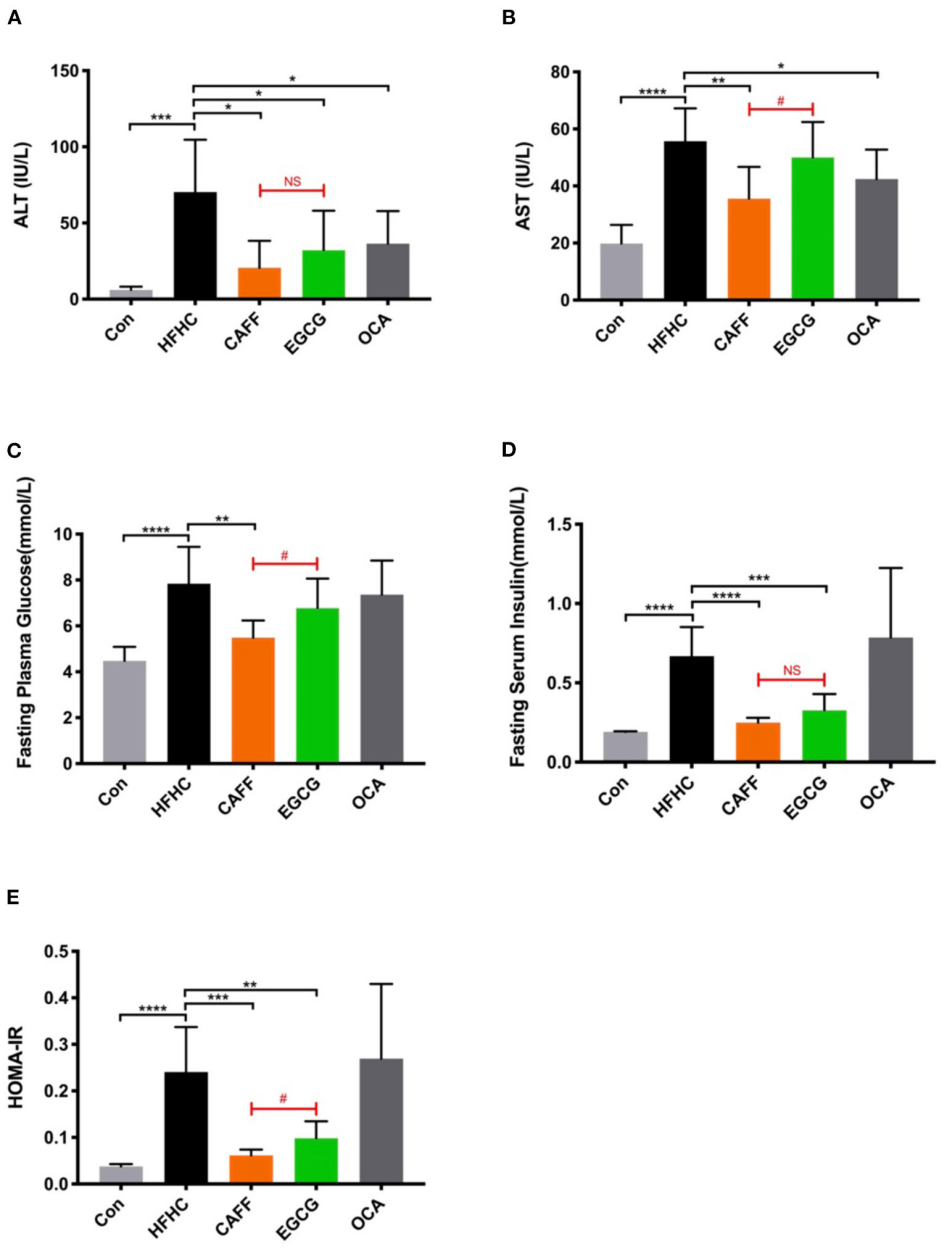
Caffeine and EGCG reverse biochemical parameters in NASH mice. Changes in serum ALT **(A)** and AST **(B)** activities, FBG **(C)**, FINS **(D)**, and HOMA-IR **(E)** of the mice from each group (*n* = 8) vs. HFHC, *P < 0.05, **P < 0.01, ***P < 0.001, ****P < 0.0001; vs. EGCG, ^#^*P* < 0.05, NS, not significant.

Next, we assessed the serum liver enzyme activity in serum and glucose metabolism levels of mice in each group to determine the pharmacodynamic differences between caffeine and EGCG treatments. After 6 weeks of treatment, the serum ALT levels of mice in each treatment group were significantly different from those in the HFHC group. There was a difference between the two treatments in their ability to reverse the increased serum AST activity caused by the HFHC diet. Caffeine significantly reversed the abnormal increase in AST caused by the HFHC diet, but there was no significant difference in AST between the EGCG and HFHC groups (Figure 3B). Additionally, caffeine significantly improved the hyperglycemia, high serum insulin, and insulin resistance index caused by the HFHC diet, whereas EGCG only reduced serum insulin and the insulin resistance index but was unable to reverse HFHC-induced hyperglycemia (Figures 3C–E). As a positive control drug, obeticholic acid can significantly reduce the levels of serum ALT and AST caused by HFHC diet, but it has no significant improvement on glucose metabolism.

### Caffeine and EGCG Markedly Decrease Hepatic Steatosis and Inflammation in a Mouse Model of NASH

**Figure 4:**
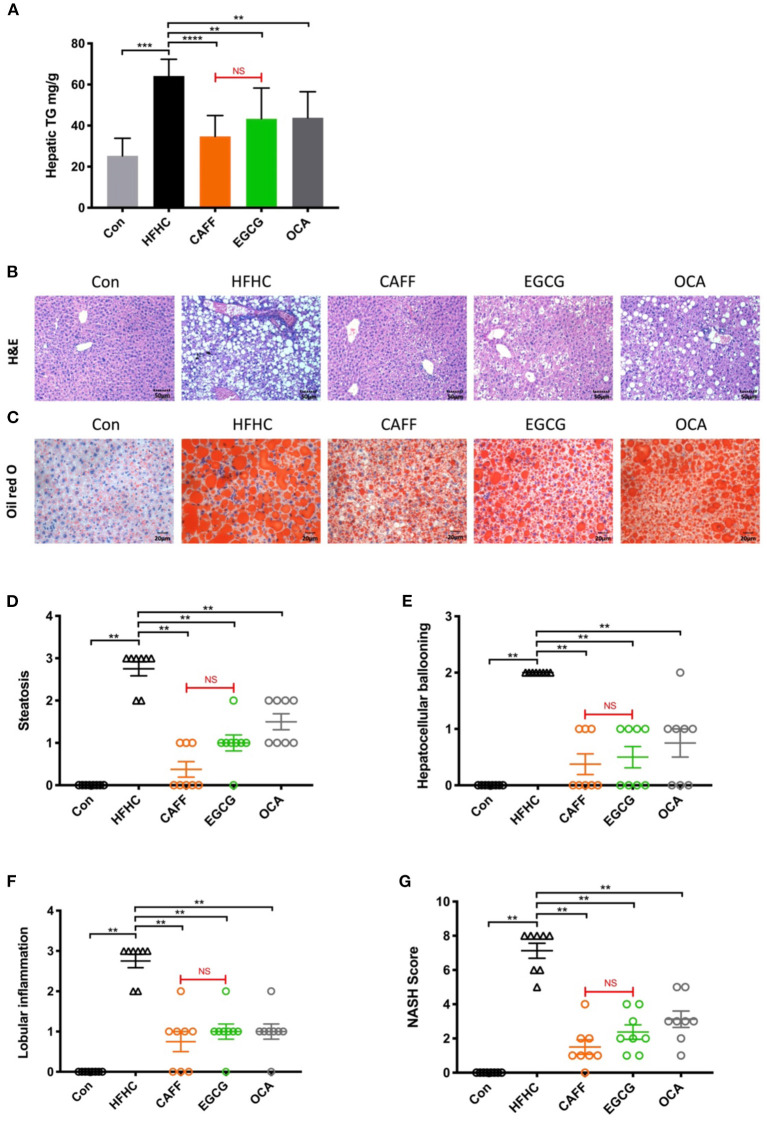
Caffeine and EGCG markedly ameliorate hepatic steatosis and inflammation in non-alcoholic steatohepatitis mice. **(A)** Hepatic TG content in mice (*n* = 8). **(B)** H&E and **(C)** Oil Red O staining for liver sections (200×). **(G)** The NAS was calculated as a sum of the scores of three parameters [steatosis **(D)**, hepatocellular ballooning **(E)**, and lobular inflammation **(F)**] vs. HFHC, **P < 0.01, ***P < 0.001, ****P < 0.0001; vs. EGCG, NS, not significant.

The hepatic steatosis and inflammation in mice with NASH were markedly attenuated after 6 weeks of caffeine or EGCG treatment. Caffeine and EGCG also reversed the abnormally elevated liver TG levels in HFHC mice, which were nearly three times higher than that in Con mice (Figure 4A). After 30 weeks of the HFHC diet, mice were observed with steatosis, ballooning of hepatocytes, and inflammatory cell infiltration in liver lobules by H&E staining. Typical macrovesicular steatosis was observed in the centrilobular regions. Oil Red O staining showed dramatically increased lipid deposition in much of the cytoplasm of hepatocytes. However, after 6 weeks of treatment with caffeine or EGCG, the steatosis of hepatocytes was reversed, and ballooning degeneration, and inflammatory infiltration were less common (Figures 4B,C). Similarly, the hepatic steatosis score (Figure 4D), balloon-like change score (Figure 4E), lobular inflammation score (Figure 4F), and total NAS score (Figure 4G) were significantly lower in the caffeine and EGCG groups than in the HFHC group. Surprisingly, there was no significant difference between the two treatments in improving hepatocyte lipid deposition or inflammation in these mice. Similarly, obeticholic acid can significantly reduce decrease hepatic steatosis and inflammation in a mouse model of NASH.

### Caffeine and EGCG Strongly Attenuate Liver Fibrosis in a Mouse Model of NASH

**Figure 5:**
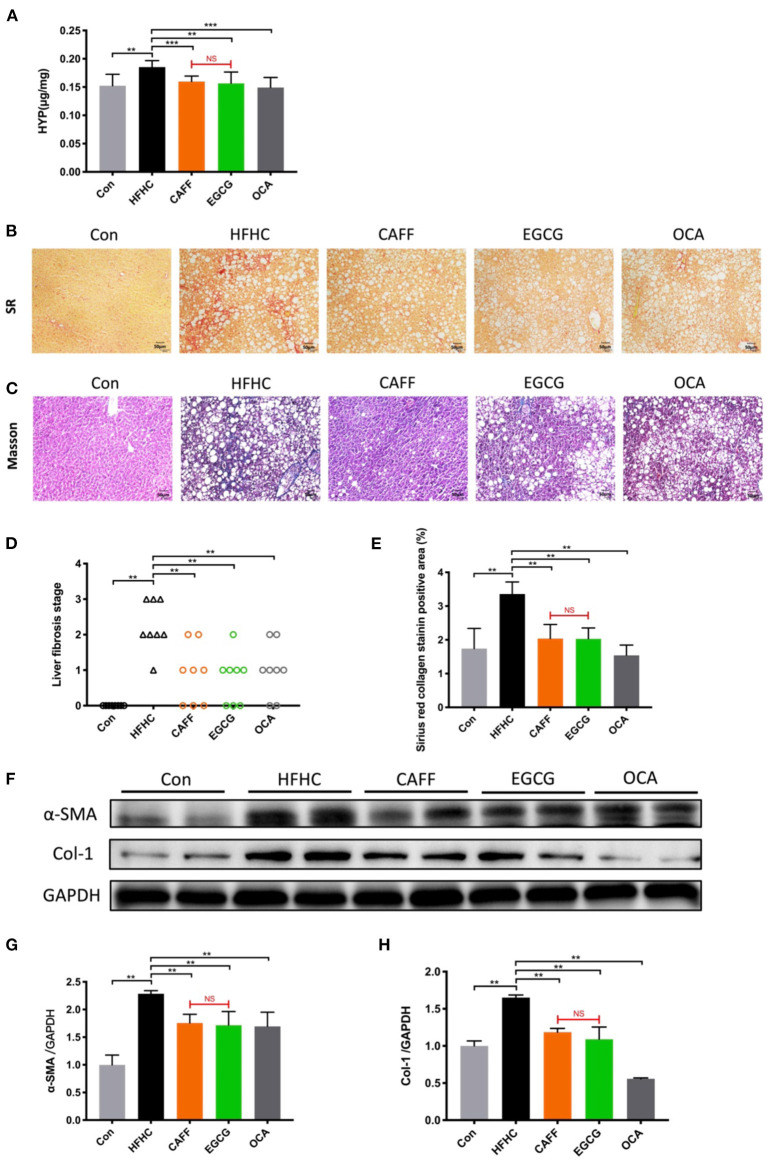
Caffeine and EGCG Strongly Attenuate liver fibrosis in NASH mice **(A)** Total liver collagen (hydroxyproline, HYP). **(B)** Sirius red staining (200×), red color indicates fibrosis. **(C)** Masson trichrome staining (200×), blue color indicates fibrosis. **(D)** Liver fibrosis stage (*n* = 8). **(E)** Liver fibrosis areas with positive collagen levels by semiquantitative analysis of Sirius red staining (*n* = 8). **(F–H)** Col-1 and α-SMA protein expression levels in liver tissue vs. HFHC, **P < 0.01, ***P < 0.001; vs. EGCG, NS, not significant.

Assessing the progression of liver fibrosis is the key to evaluating the therapeutic potential of NASH-related drugs (38); therefore, the extent of liver fibrosis in mice of each group was evaluated. After 30 weeks of the HFHC diet, the HYP content in the liver tissue of mice in the HFHC group was significantly higher than that of mice in the Con group. However, after 6 weeks of caffeine or EGCG treatment, the HYP content in the liver decreased significantly, and there was no significant difference between the two treatments (Figure 5A). Sirius Red staining of the liver tissue indicated that the hepatocytes in the Con group were arranged in an orderly fashion, the structure of the liver lobules was clear, and the hepatocyte morphology was normal. Conversely, the liver tissue structure of the HFHC group was destroyed and replaced by a large amount of proliferative fibrous tissue. The fibrous tissue in the caffeine and EGCG groups were decreased compared to the HFHC group (Figure 5B). Masson staining showed results similar to those of Sirius Red staining. There was no significant difference in the improvement of liver fibrosis in the caffeine and EGCG groups (Figure 5C). The fibrosis stage was determined by combining two pathological staining images from each liver (*n* = 8). In the HFHC group, nearly 40% of the mice reached the F3 stage, and 50% of the mice reached the F2 stage. In contrast, the degree of liver fibrosis decreased significantly after administration of caffeine and EGCG, and nearly 80% of the mice had F1-F2 stage fibrosis (Figure 5D). The positive staining area of fibrosis in the HFHC group was significantly higher than that in the Con group, which was consistent with the pathological findings. The positive staining areas of fibrosis in the caffeine and EGCG groups were similar to that in the Con group (Figure 5E).

α-SMA is a marker of hepatic stellate cell activation, and Col-1 is the main component of hepatic collagen fibers. These two indexes are of great significance in determining the degree of hepatic fibrosis. α-SMA and Col-1 were highly expressed in liver tissues from the HFHC group compared to those in the Con group. After treatment with caffeine or EGCG, α-SMA and Col-1 protein levels decreased significantly. There was no significant difference between the two treatments (Figures 5F–H). As a positive control drug, OCA can be strongly attenuate liver fibrosis in a mouse model of NASH.

### Caffeine and EGCG Drastically Changed the Hepatic Gene Expression in a Mouse Model of NASH, and the Regulation Range of Caffeine Was Wider Than That of EGCG

**Figure 6:**
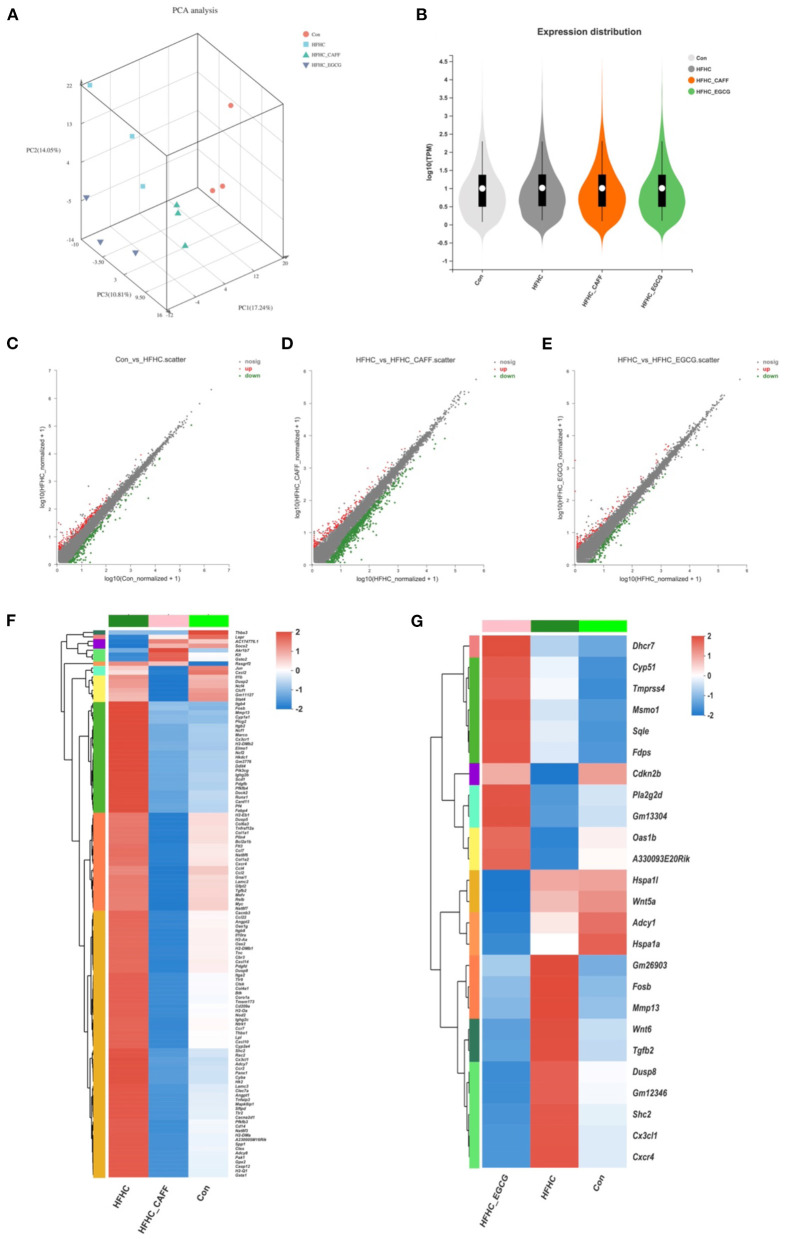
Transcriptome characterization of liver tissue. **(A)** Transcriptomics PCA plots. Red circles indicate Con group, blue squares indicate HFHC group, light green triangles indicate CAFF group, purple triangles indicate EGCG group. **(B)** Violin plots show median (white point), quartiles (black bars), and kernel density estimation (violin) for each score distribution. Scatter plot analysis, Con vs. HFHC **(C)**, HFHC vs. CAFF **(D)**, HFHC vs. EGCG **(E)**. **(F)** Clustering heat map of HFHC vs. CAFF differential gene expression. **(G)** Clustering heat map of HFHC vs. EGCG differential gene expression.

We selected three liver tissue samples each from the Con, HFHC, caffeine treated, and EGCG treated groups for transcriptome microarray analysis, to explore the pharmacological mechanisms of caffeine and EGCG in reversing the pathological states of lipid deposition, inflammation, glucose metabolism, and fibrosis. Principal component analysis showed that the 12 samples could be clearly assigned to four groups (Con, HFHC, HFHC_CAFF, and HFHC_EGCG; Figure 6A). Violin plots denote the expression distribution of candidate genes. The gene distributions of the four groups of mice were similar, indicating that the samples were qualified, and no mutant genes appeared (Figure 6B). Next, we screened candidate genes, using log FC > 2 and *p* < 0.05 to identify differentially expressed genes. We examined the differential gene sets between the Con group and the HFHC group (Con vs. HFHC), between the HFHC group and the caffeine group (HFHC vs. HFHC_CAFF), and between the HFHC group and the EGCG group (HFHC vs. HFHC_EGCG). The three differential gene sets were analyzed using a scatter plot. There were 1,477 differentially expressed genes between the Con group and the HFHC group, including 1,135 upregulated and 342 downregulated genes (Figure 6C). There were 764 differentially expressed genes between the HFHC group and the caffeine group, including 147 upregulated and 617 downregulated genes (Figure 6D). Lastly, there were 218 differentially expressed genes between the HFHC group and the EGCG group, including 103 upregulated and 115 downregulated genes (Figure 6E).

To examine genes and pathways that may be specifically regulated by caffeine and EGCG in the context of NASH, we selected genes that were differentially expressed in mice receiving the HFHC diet compared to the control diet and genes that were differentially expressed in mice receiving caffeine, which we termed the Caffeine Target Gene Set (Con vs. HFHC and HFHC vs. HFHC_CAFF), for cluster analysis and created a heat map (Figure 6F). Similarly, we selected genes that were differentially expressed in mice receiving the HFHC diet compared to the control diet and genes that were differentially expressed in mice receiving EGCG, which we termed the EGCG Target Gene Set (Con vs. HFHC and HFHC vs. HFHC_EGCG), for cluster analysis and created a heat map (Figure 6G). The heat maps of the target gene clusters showed that the number and overall fold-change of caffeine-regulated genes in the NASH disease background was significantly higher than those of EGCG-related genes (1,208 vs. 294), suggesting that caffeine regulates a wider range of genes than EGCG in this NASH mouse model.

### Functional Enrichment Analysis of NASH Regulatory Mechanisms by Caffeine and EGCG

**Figure 7:**
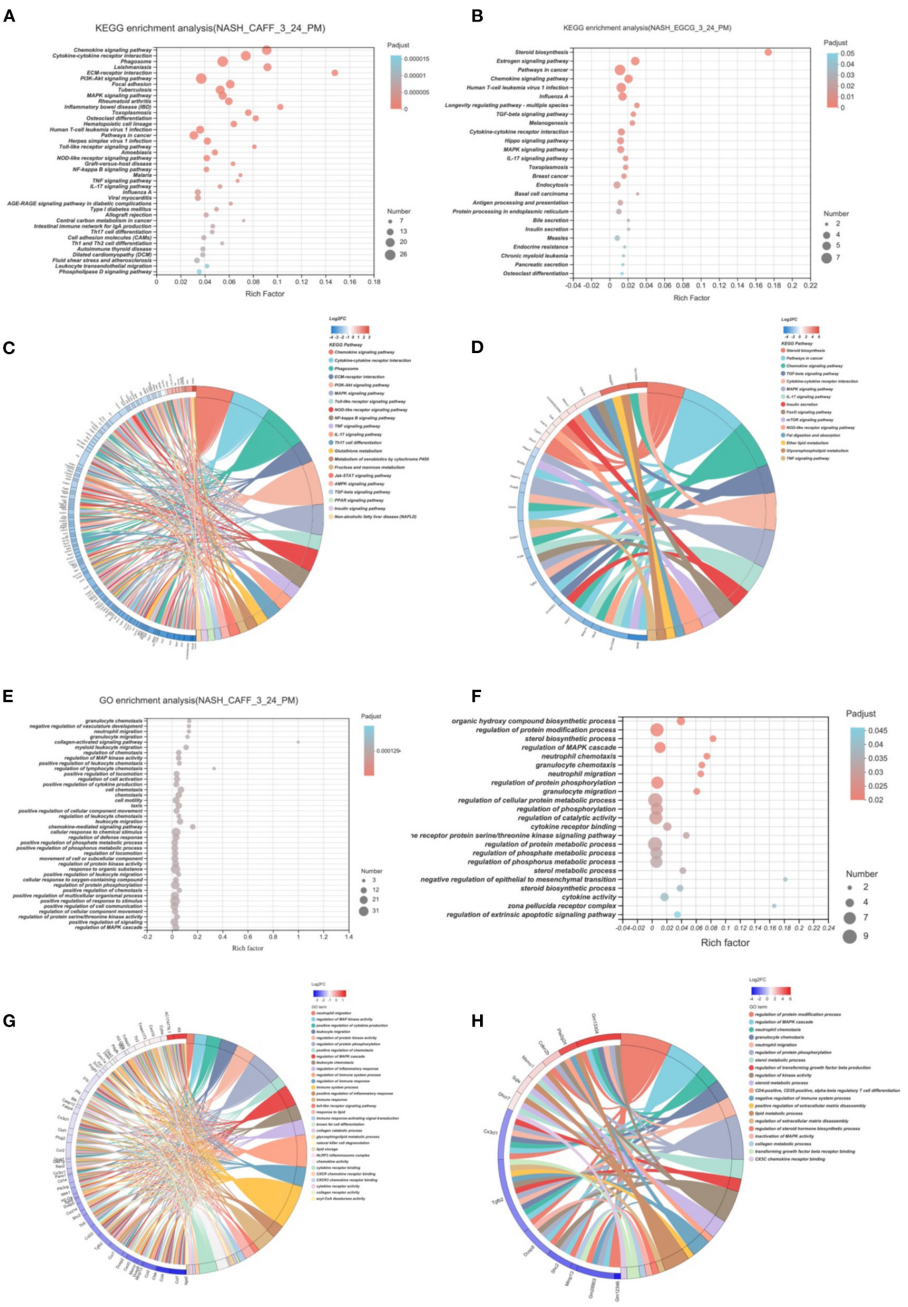
Functional Enrichment Analysis of Caffeine and EGCG Gene Sets. Bubble graph of KEGG enrichment analysis of different genes of caffeine **(A)** and EGCG **(B)** compared with HFHC. KEGG enrichment analysis of caffeine **(C)** and EGCG **(D)** differential genes and enrichment circos graph of NASH related pathways. Bubble graph of GO enrichment analysis of different genes of caffeine **(E)** and EGCG **(F)** compared with HFHC. KEGG enrichment analysis of caffeine **(G)** and EGCG **(H)** differential genes and enrichment circos graph of NASH related pathways.

To study the functional changes after caffeine and EGCG treatment, we performed KEGG pathway analysis and GO functional enrichment analysis on genes that were differentially expressed after treatment with caffeine (caffeine vs. HFHC) or EGCG (EGCG vs. HFHC). Representative highly enriched KEGG pathways from the caffeine and EGCG groups are shown using bubble plots (Figures 7A,B). The targets of caffeine and EGCG overlapped significantly with the pathological pathways involved in NASH. To further investigate the effect of caffeine and EGCG in the treatment of NASH, we selected major pathways involved in NASH and assessed the detailed relationships between differentially expressed genes (log_2_ FC > 2) using a Circos graph (Figures 7C,D). Pathway analysis classified genes regulated by caffeine as being involved in chemokine signaling, cytokine-cytokine receptor interaction, phagosome, ECM-receptor interaction, PI3K-AKT signaling, MAPK signaling, Toll-like receptor signaling, NOD-like receptor signaling, NF-kappa B signaling, and others. Similarly, pathway analysis classified genes regulated by EGCG as being involved in steroid biosynthesis, pathways in cancer, chemokine signaling, TGF-beta signaling, cytokine-cytokine receptor interaction, MAPK signaling, IL-17 signaling, insulin secretion, FoxO signaling, and mTOR signaling.

After analyzing the pathways that caffeine and EGCG affect in our NASH model, we analyzed the GO function enrichment of differentially regulated genes and analyzed the biological processes that the two treatments regulate in the context of NASH. Representative highly enriched biological process clusters from the caffeine and EGCG groups are shown in bubble plots. GO functional enrichment analysis showed that caffeine-related differentially expressed genes were significantly enriched in granulocyte chemotaxis, neutrophil migration, granulocyte migration, collagen-activated signaling, regulation of MAP kinase activity, positive regulation of cytokine production, and other functions (Figure 7E). EGCG-related differentially expressed genes were significantly enriched in the regulation of protein modifications, sterol biosynthesis, regulation of the MAPK cascade, neutrophil chemotaxis, granulocyte chemotaxis, neutrophil migration, and other functions (Figure 7F). These biological functions are similar to those altered during the development of NASH, such as energy metabolism and fibrosis. To further investigate the mechanisms behind the effects of caffeine and EGCG in the treatment of NASH, we focused on clusters highly correlated with NASH and analyzed the expression levels of specific genes in these clusters using a heatmap Circos graph (Figures 7G,H). Functional analysis classified genes that were differentially regulated by caffeine as being involved in lipid storage, chemokine activity, collagen receptor signaling, immune response, and acyl-CoA desaturase activity. Similarly, pathway analysis classified genes differentially regulated by EGCG as being involved in sterol metabolic processes, lipid metabolic processes, regulation of the MAPK cascade, and CX3C chemokine receptor binding. This suggests that the biological functions of caffeine and EGCG may significantly overlap with NASH-related pathological processes, such as lipid deposition, inflammation, and fibrosis progression.

### Potential Pharmacological Mechanisms of Caffeine and EGCG in the Treatment of NASH Highly Coincide With the Inflammatory Pathway, but Each Has Its Own Emphasis

**Figure 8:**
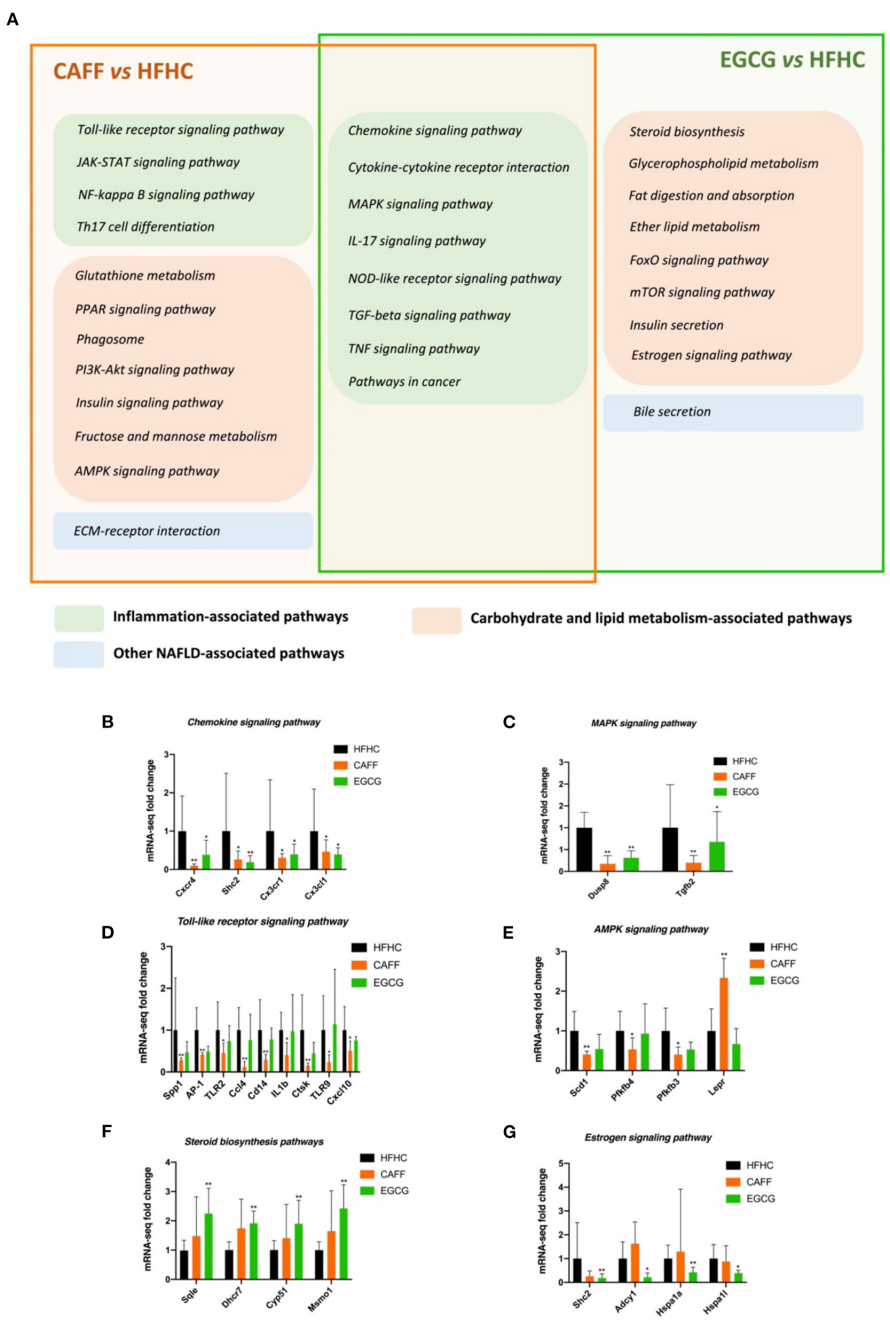
The similarities and differences of the difference pathway between CAFF and HFHC and the difference pathway between EGCG and HFHC **(A)**. The mRNA-seq expression of differential genes in chemokine signaling pathway **(B)**, MAPK signaling pathway **(C)**, Toll-like receptor signaling pathway **(D)**, AMPK signaling pathway **(E)**, Steroid biosynthesis signaling pathway **(F)**, Estrogen signaling pathway **(G)**. vs. HFHC, *P < 0.05, **P < 0.01.

Next, we analyzed and classified the enriched pathways of caffeine and EGCG in the context of NASH and examined the similarities and differences in the pharmacological mechanisms of the two treatments. As shown in Figure 8A, the pharmacological signaling pathways of the two treatments in the context of NASH were mostly distributed in the inflammatory signaling pathway and the glycolipid metabolism signaling pathway. Interestingly, the two drugs both strongly affected eight inflammation-related signaling pathways: chemokine signaling, cytokine-cytokine receptor interaction, MAPK signaling, IL-17 signaling, NOD-like receptor signaling, TGF-beta signaling, TNF signaling, and pathways in cancer (Figure 8A, the intersection of the orange box and green box). Caffeine regulated four inflammation-related signaling pathways (Toll-like receptor signaling, JAK-STAT signaling, NF-kappa B signaling, and Th17 cell differentiation) and seven metabolic signaling pathways (glutathione metabolism, PPAR signaling, phagosome, PI3K-Akt signaling, insulin signaling, fructose and mannose metabolism, and AMPK signaling) that were not regulated by EGCG (Figure 8A, left side of orange box). In contrast, EGCG-specific pharmacological pathways were almost entirely metabolic signaling pathways, including steroid biosynthesis, glycerophospholipid metabolism, fat digestion and absorption, ether lipid metabolism, FoxO signaling, mTOR signaling, insulin secretion, and estrogen signaling. Although caffeine and EGCG both regulated the metabolic pathway, caffeine had a greater effect on glucose metabolism, whereas EGCG had a greater effect on lipid metabolism.

Next, we selected some common and specific pharmacological pathways affected by caffeine and EGCG for differential gene mRNA level tracing. For example, the chemokine and MAPK signaling pathways are representative of the common pharmacological pathways. The mRNA levels of key genes in these pathways that were regulated by both drugs, such as *Cxcr4, Shc2, Cx3cr1, Cx3cl1*, and *Dusp8*, were significantly different in treated mice than in HFHC mice (Figures 8B,C). The toll-like receptor signaling pathway and glutathione metabolism are representative of caffeine-specific pharmacological pathways. The mRNA levels of key genes in these pathways, such as *Spp1, AP-1, TLR2, Ccl4, IL1b, Scd1*, and *Lepr*, were significantly different in caffeine-treated mice and HFHC mice (Figures 8D,E). Steroid biosynthesis and the estrogen signaling pathway are representative of EGCG-specific pharmacological pathways. The mRNA levels of key genes in these pathways, such as *Sqle, Dhcr7, Cyp51, Shc2, Hspa1a*, and *Hspa1l*, were significantly different between EGCG-treated mice and HFHC mice as well (Figures 8F,G).

## Discussion

In this study, we evaluated the protective effects and mechanism of action of caffeine and EGCG treatments against HFHC diet-induced NASH in mice. Caffeine and EGCG are the main active substances in coffee and tea, respectively, which are the most popular drinks in the western and eastern regions. Our previous study confirmed that an HFHC diet can induce NASH in mice (37). Using this model, we focused on observing changes in hepatic lipid deposition, inflammation, and fibrosis in the presence or absence of caffeine or EGCG in this NASH model. We then compared the effects of the two treatments on these pathological processes. Although there was no significant difference between the two treatments in improving these pathological features of NASH, the overall benefit trend of caffeine was greater than that of EGCG. To better understand the potential therapeutic mechanisms of caffeine and EGCG, we combined transcriptome microarray, KEGG enrichment analysis, and GO enrichment analysis to identify the potential pharmacological effects of caffeine and EGCG against HFHC-induced NASH. Finally, these possible pharmacological mechanisms were analyzed and integrated to explore the commonality and specificity of caffeine and EGCG against NASH in mice fed an HFHC diet. Both drugs significantly reversed the abnormal regulation of multiple biological processes in NASH, but the pharmacological mechanism of caffeine to improve NASH was more extensive than that of EGCG. The two drugs may greatly overlap in improving NASH inflammation, but caffeine may have better potential in regulating glucose metabolism whereas EGCG may have better potential in regulating lipid metabolism.

Increasing evidence shows that caffeine and EGCG have wide range of biological effects that can be used for the prevention and treatment of NASH. Convincingly, the Chinese guidelines for the prevention and treatment of NAFLD regard drinking more coffee and tea as a C1 recommendation (39). Growing epidemiological and clinical evidence indicates that consumption of coffee and green tea reduces the severity of NASH. In an Italian study, 137 patients with NAFLD and 108 controls were enrolled, and coffee intake was determined by the absolute number of cups of coffee consumed. Compared with non-coffee drinkers, those who consumed coffee had less severe fatty liver disease (40). A case-control study from Mexico also found similar protective effects of coffee against NAFLD, as assessed by ultrasonography (41). Similarly, patients with NASH who were diagnosed by a liver biopsy and took 600 mg of green tea catechins (including 52.6% EGCG) per day for 6 months with controlled diets and exercise therapy achieved significant effects, including improved anthropometric parameters and biochemistry levels (42). However, not all evidence showed clinical efficacy, which could be related to the different doses, formulation issues, or duration of studies. In a recent report by Mansour administering caffeine to diabetic NAFLD patients' results are negative and do not support the administration of caffeine (43). Therefore, in this study, we carefully considered the intervention dose (described in detail in the next paragraph) and intervention time of caffeine and EGCG, so as to provide a reference for the disease degree and intervention dose selection of these two substances in future clinical research.

We expect that our results may be relevant for humans with NASH. The chosen caffeine dose in this study (75 mg/kg per day) is equivalent to 6 mg/kg per day in humans. It is estimated that an adult human (mean weight, 70 kg) with moderate coffee intake consumes approximately 400 mg of caffeine per day, which is 6 mg/kg per day (44). Furthermore, 6 mg/kg caffeine is equivalent to three cups of coffee (45). The average green tea drinker could be defined as a 60 kg man who consumes 10 g of green tea in four cups every day. A typical cup of green tea, brewed using 2.5 g of tea leaves, contains 620–880 mg of water-extractable materials, of which EGCG accounts for ~16.67–25% (46). Considering the differences in body surface area, the optimum EGCG dose for mice was 100 mg/kg/d. See Figure 1D for the conversion between the dosage of mice and the daily consumption of humans, which is calculated according to the body surface area.

Although there are many studies on caffeine and EGCG, there are few multilevel horizontal comparisons between the two drugs, as treatments. We administered 75 mg/kg caffeine and 100 mg/kg EGCG to observe the commonality and specificity of the two treatments in an HFHC-induced mouse model of NASH. We first compared the differences between these two compounds in improving the phenotype of mice with NASH. In general, the body shapes of mice in the caffeine and EGCG groups were significantly more normal than those of mice in the HFHC group, and the weights were lower. Furthermore, the body weights of mice in the caffeine group were lower than those of mice in the EGCG group. This difference occurred by the 4th week (28 weeks) of drug administration (Figure 2C). We believe that this difference may be related to the ability of caffeine to inhibit appetite, as it has neuroexcitatory effects (47). Consistent with the trend in body weight, the liver weight, liver/body ratio, and spleen weight in the caffeine and EGCG groups were significantly lower than those in the HFHC group, and the liver morphologies were more normal. The effect of the two drugs was significantly different only in terms of the spleen weight. NASH involves a range of pathological conditions in the liver, including steatosis, inflammation, hepatocyte injury, and fibrosis, and is accompanied by systemic insulin resistance (48). Therefore, we focused on the ability of caffeine and EGCG to improve these important pathological features of NASH. Caffeine normalized the elevated ALT and AST levels caused by the HFHC diet, but EGCG reduced only ALT levels and had no significant effect on AST (Figures 3A,B). These results are consistent with those observed in earlier studies (49, 50). In terms of systemic insulin resistance, both caffeine and EGCG significantly reduced the HOMA-IR index and fasting insulin level, although caffeine was better than EGCG at reducing the HOMA-IR index. Unlike caffeine, EGCG did not significantly reduce fasting blood glucose levels. Lipid accumulation and inflammation in hepatocytes are key pathological features of NAFLD. Prior studies have noted that coffee and tea can significantly improve liver lipid deposition and inflammation in NASH patients and animal models (51, 52). Consistent with the literature, we found that caffeine and EGCG significantly improved lipid deposition and inflammation in an HFHC-induced NASH mouse model, without a significant difference between the two treatments. This result may be explained by the fact that both coffee and tea strongly regulate fat metabolism and inflammation-related targets. Several recent clinical studies showed that coffee and tea are associated with the improvement of liver fibrosis and cirrhosis in patients with NASH (13, 53). Our results also illustrate the role of caffeine and EGCG in the improvement of liver fibrosis. Caffeine and EGCG were also able to reverse the effects of the HFHC diet on the liver HYP, liver fibrosis, and the expression of liver fibrosis proteins. The degrees of reversal were similar. We believe that the commonality and specificity of the two treatments are the reasons for these differential effects in NASH.

The transcriptome represents a link between the genetic information encoded in the genome and the biological function of the proteome. Regulation at the transcriptional level is the most important pattern of self-regulation. Therefore, we examined the transcriptomes of these mouse liver tissue samples using a transcriptome microarray. The results indicated that after drug intervention, the number of differentially expressed genes between the caffeine and HFHC groups was more than three times that which was observed between the EGCG and HFHC groups. There were 764 differentially expressed genes between the caffeine and HFHC groups, whereas there were only 218 differentially expressed genes between the EGCG and HFHC groups, indicating that drug therapy affected multiple genes. This shows that more biological reactions in the liver of mice with NASH were stimulated after caffeine treatment than after EGCG treatment. These differentially expressed genes are likely to be potential targets of caffeine and EGCG in mice with NASH. The functional classification of these differentially expressed genes can help us better determine the potential pharmacological mechanisms of the two drugs.

Next, KEGG signal pathway enrichment analysis and GO functional enrichment analysis of the two groups of DEGs were performed. Caffeine was involved in more KEGG signaling pathways than EGCG (Figures 7A,B). Twenty caffeine-involved signaling pathways were related to NASH, such as chemokine signaling, cytokine-cytokine receptor interaction, phagosome, ECM-receptor interaction, PI3K-AKT signaling, MAPK signaling, Toll-like receptor signaling, NOD-like receptor signaling, and NF-κB signaling. Classification of these pathways according to the involved pathological process revealed a broad division into inflammation-related pathways (chemokine signaling, cytokine-cytokine receptor interaction, MAPK signaling, Toll-like receptor signaling, NOD-like receptor signaling, NF-κB signaling, etc.), metabolism-related pathways (phagosome, PI3K-AKT signaling, etc.), and fibrosis-related pathways (ECM-receptor interaction) (Figure 7C). Interestingly, these pathological processes were consistent with the GO functional enrichment analysis. GO analysis showed that caffeine was involved in the biological processes of inflammation (neutrophil migration, regulation of MAP kinase activity, immune system process, natural killer cell degranulation, etc.), metabolism (response to lipid, glycosphingolipid metabolic process, lipid storage, etc.), and fibrosis (collagen receptor activity, etc.) (Figures 7E,G). This is consistent with previous research showing that caffeine or coffee can improve NASH-related pathological processes, such as lipid deposition, insulin resistance, oxidative stress, and fibrosis (29, 54, 55).

EGCG was associated with fewer enriched KEGG pathways than caffeine, including inflammation-related pathways (chemokine signaling, cytokine-cytokine receptor interaction, MAPK signaling, Toll-like receptor signaling, NOD-like receptor signaling, NF-κB signaling, etc.), metabolism-related pathways (steroid biosynthesis, glycerophospholipid metabolism, fat digestion and absorption, mTOR signaling, insulin secretion, FoxO signaling, etc.), and other NAFLD-associated pathways (bile secretion) (Figure 7D). GO analysis showed that EGCG affected genes involved in inflammation (regulation of MAPK cascade, neutrophil migration, transforming growth factor beta receptor binding, CD4-positive CD25-positive alpha-beta regulatory T cell differentiation, etc.), metabolism (response to lipid, glycosphingolipid metabolic process, lipid storage, etc.), and fibrosis (collagen receptor activity, etc.) (Figures 7F,H). These results are consistent with those of Kochi and Xiao, who suggested that EGCG reduces the severity of liver injury in an experimental model of NAFLD and is associated with lower concentrations of pro-fibrogenic molecules, reactive oxygen species, and pro-inflammatory mediators by modulating TGF/SMAD, PI3K/Akt/FoxO1, and NF-κB signaling (28, 56).

One of the aims of this study was to determine the differences in pharmacological mechanisms between caffeine and EGCG. Therefore, we compared the transcriptome data for the two treatments. We found a high degree of overlap for both GO functional enrichment analysis and KEGG signal pathway enrichment analysis, including inflammation-related biological functions and signaling pathways. Among them, the overlap in inflammatory signaling pathways in the KEGG analysis was the most obvious; a total of eight signaling pathways overlapped (chemokine signaling, cytokine-cytokine receptor interaction, MAPK signaling, IL-17 signaling, NOD-like receptor signaling, TGF-beta signaling, TNF signaling, and pathways in cancer). We speculate that this overlap may be the reason for the similarity in the effects of the two treatments. However, the two treatments also had their own uniquely involved pathways, which may cause some of the differential effects. Caffeine affected more inflammatory pathways than EGCG, suggesting that caffeine is more involved in inflammation-related pathways. Both drugs affected genes that were enriched in metabolic pathways, but caffeine was more involved in the glucose metabolic pathway, whereas EGCG was more involved in the lipid metabolic pathway.

We further explored the target genes in the transcriptome data and identified multiple genes that may serve as downstream targets of therapy for NASH. We selected representative genes in the overlapping pathways of the two treatments (chemokine signaling and MAPK signaling) and pathways specific to the two treatments (Toll-like receptor signaling, AMPK signaling, steroid biosynthesis, and estrogen signaling). As a chemokine receptor, CXCR4 has a strong chemotactic effect on immune cells and plays an important role in the occurrence and development of NASH (57). Caffeine and EGCG significantly reduced the abnormal expression of *Cxcr4, Cx3cr1, Shc2*, and *Cx3cl1* induced by an HFHC diet. Similarly, caffeine and EGCG exhibited significant inhibitory effects on *Dusp8* and *TGF-*β*2*, factors in the MAPK pathway that activates protein activity in cascades during the development of NASH (58). We selected a representative pathway from the inflammatory and metabolic pathways enriched by either caffeine or EGCG for gene expression traceability. *AP-1, TLR2*, and *IL1-*β, genes that are differentially regulated during the pathological progression of NASH liver inflammation, and *SCD1, LEPR*, and other genes related to NASH lipid deposition, were regulated by caffeine, but EGCG had no significant regulatory effect. Conversely, EGCG affected the expression of *Sqle, CYP51, Hspa1a*, and other genes related to NASH metabolism, but caffeine did not show a significant regulatory effect.

Last but not the least, there are two main strengths in this study. First, we intervened with caffeine and EGCG to compare and observe the protective effects of the two natural products in the liver of mice under the background of overnutrition. Second, we established the mouse transcriptome bioinformatics database of these two natural products under the background of overnutrition and made a comparative analysis from the possible pharmacological mechanisms, which provided more comprehensive data for other researchers.

OCA is an agonist of FXR (Farnesoid X receptor). It mainly exerts pharmacological effects by activating FXR. It was originally a new generation drug for the treatment of primary biliary cirrhosis. Recent studies have found that OCA shows potential value in the treatment of NAFLD, especially NASH (59). Therefore, we used OCA as a positive control drug in this study. In this study, OCA can also significantly reduce the content of TG and hydroxyproline in the liver tissue of the model mice. The histological changes show that the degree of steatosis and liver fibrosis of OCA group mice are reduced, which is consistent with the experimental results of previous researchers.

However, in our current study, we only describe preliminary framework analyses of caffeine and EGCG in the context of NASH and do not further explore or verify the differential genes and pathways.

In this study, the HFHC-induced NASH mouse model was used to observe and compare caffeine and EGCG, the active components of coffee and tea, respectively, in the context of NASH. We examined their effect on the phenotype and the possible pharmacological mechanisms, and a daily intake dose close to clinical humans was used. This provides a solid research foundation for both clinical researchers and basic researchers to further explore the clinical application or pharmacological mechanism of caffeine and EGCG in the future.

## Conclusion

In summary, this study confirmed that 75 mg/kg caffeine and 100 mg/kg EGCG (human caffeine and EGCG consumption converted into mice doses) could significantly improve liver lipid deposition, glucose metabolism, inflammation, and fibrosis in a mouse model of NASH induced by an HFHC diet. Although there was no significant difference between the two treatments in improving these pathological features, the overall benefit trend of caffeine was greater than that of EGCG. Both drugs affected abnormal gene expression in NASH, but the pharmacological mechanism of caffeine was more extensive than that of EGCG. The two drugs may greatly overlap in improving the mechanisms driving NASH progression, but caffeine may have better potential in regulating glucose metabolism, whereas EGCG may have better potential in regulating lipid metabolism.

## Data Availability Statement

The original contributions presented in the study are publicly available. This data can be found here: https://www.ncbi.nlm.nih.gov/bioproject/PRJNA772645.

## Ethics Statement

The animal study was reviewed and approved by Animal Experiment Ethics Committee of Shanghai University of traditional Chinese Medicine.

## Author Contributions

XX completed the animal husbandry, biochemical experiments, data analysis of this research, and wrote the manuscript. CC partly participated in the design of the research study and critically discussed and revised the manuscript. CB-y was responsible for the animal husbandry of this study and the completion of certain biochemical tests. LH-s and TH-j provided some experimental technical expertise in this study. WX, SQ-m, and AZ-m assisted in processing the analysis of some experimental data in this study. HY-y was responsible for the design of the experimental protocol and the writing of the experimental part of the study. FQ was responsible for the planning of the overall experimental scheme and participated in the discussion, revision, completion of the final manuscript, and most of the experiments in this study. All authors read and approved the final manuscript.

## Funding

This work was supported by the Shanghai Science and Technology Development Funds (No. 18401933100 to FQ), the Shanghai Shenkang three-year action plan (No. SHDC2020CR4051 to FQ), and the National Natural Science Foundation of China (No. 82174040 to FQ; No. 81830119 to HY-y).

## Conflict of Interest

The authors declare that the research was conducted in the absence of any commercial or financial relationships that could be construed as a potential conflict of interest.

## Publisher's Note

All claims expressed in this article are solely those of the authors and do not necessarily represent those of their affiliated organizations, or those of the publisher, the editors and the reviewers. Any product that may be evaluated in this article, or claim that may be made by its manufacturer, is not guaranteed or endorsed by the publisher.
