# Supplementary material for: Caffeine and EGCG Alleviate High-Trans Fatty Acid and High-Carbohydrate Diet-Induced NASH in Mice: Commonality and Specificity
Source: Front Nutr. 2021 Nov 22;8:784354. doi: 10.3389/fnut.2021.784354 (PMC8647766; doi:10.3389/fnut.2021.784354)
Supplement: Supplementary file 1 [file Presentation_1.PPTX]

## Slide 1
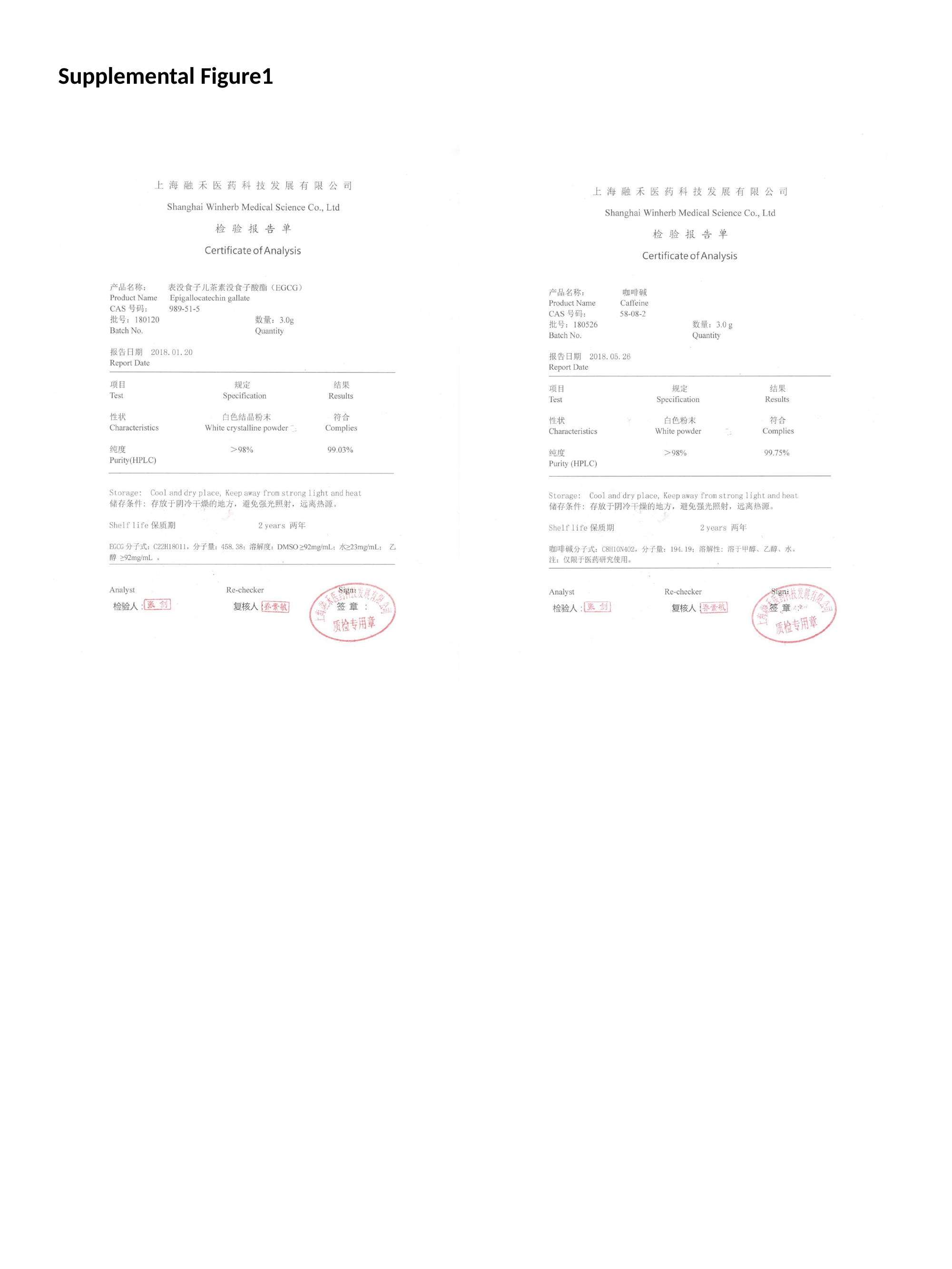

Supplemental Figure1

## Slide 2
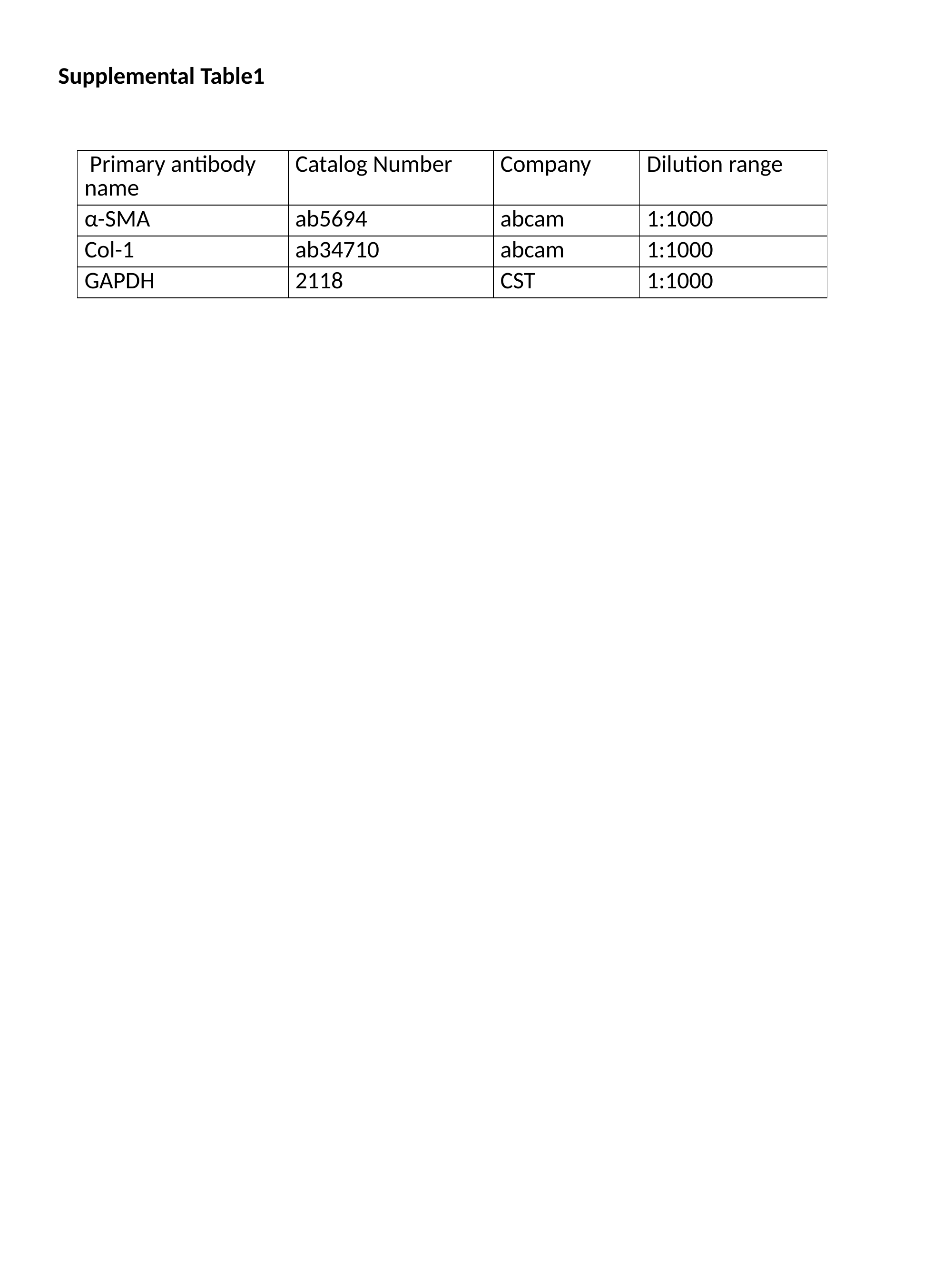

Supplemental Table1
| Primary antibody name | Catalog Number | Company | Dilution range |
| --- | --- | --- | --- |
| α-SMA | ab5694 | abcam | 1:1000 |
| Col-1 | ab34710 | abcam | 1:1000 |
| GAPDH | 2118 | CST | 1:1000 |
